# Predictors of short- and long-term mortality among acutely admitted older patients: role of inflammation and frailty

**DOI:** 10.1007/s40520-021-01926-8

**Published:** 2021-07-13

**Authors:** Michela Zanetti, Giovanna Marzaro, Paolo De Colle, Gabriele Toigo, Dario Bianchini, Mariapaola Nastri, Cristina Suriano, Rocco Barazzoni, Gianfranco Sanson

**Affiliations:** 1 Department of Medical, Surgical and Health Sciences, University of Trieste, Strada di Fiume 447, 34100 Trieste, Italy; 2 Department of Internal Medicine, University Hospital, Trieste, Italy

**Keywords:** Glasgow Prognostic Score, Inflammation, Multidimensional Prognostic Index, Osmolarity, Mortality, Hospital

## Abstract

**Background:**

Frailty, demographic and clinical variables linked to incident diseases (e.g., dehydration, inflammation) contribute to poor outcomes in older patients acutely hospitalized. Their predictivity on short-, intermediate- and long-term mortality in a comprehensive model has been scarcely investigated.

**Aims:**

To test the performance of a predictive tool considering frailty and inflammation as well as age, sex and impaired hydration status on 1-year mortality in acutely admitted older patients.

**Methods:**

Retrospective observational study including 529 medical patients (age 84.6 ± 7.3 years). At hospital admission, frailty was assessed by the Multidimensional Prognostic Index (MPI). The Glasgow Prognostic Score (GPS) was used to grade systemic inflammation. Serum osmolarity was calculated to assess hydration.

**Results:**

After adjusting for age, sex, GPS and osmolarity, the severe-risk MPI was a strong predictor for 1-year mortality (OR 4.133; 95% CI 2.273–7.516; *p* < 0.001). Age > 85 years, male sex, GPS-2 and serum osmolarity > 300 mOsm/L were independent predictors of mortality in the same multivariable model. The MPI alone showed a moderate discrimination power (AUC 0.678; 95% CI 0.628–0.729; *p* < 0.001) on 1-year mortality, which increased by 12.5% after the addition of the above predictors in the fully adjusted regression model (AUC 0.763; 95% CI 0.719–0.807; *p* < 0.001). The severe-risk MPI adjusted for the same factors was also an independent predictor of mortality after 60 and 180 days since hospital admission.

**Discussion:**

Inflammation and impaired hydration are potentially modifiable risk factors for severe outcomes in older acutely hospitalized patients. A model combining GPS, age, gender, and plasma osmolarity improved the accuracy of MPI at admission in predicting long-term mortality.

## Introduction

The need for hospital admission due to acute medical conditions increases with age as well as the rate of morbidity and mortality [1]. The risk of prolonged hospital stay, developing geriatric syndromes, institutionalization and death is higher in older frail patients [2] making diagnosis and scoring severity of frailty an essential requisite to guide decision making and treatment plan [3, 4]. The Multidimensional Prognostic Index (MPI), derived from the Comprehensive Geriatric Assessment (CGA), includes information on functional, nutritional, cognitive and social status, polypharmacy and comorbidities. The MPI has proved to represent an excellent instrument to predict short- and long-term adverse clinical outcomes including mortality in older frail patients affected by several diseases [5, 6].

In addition to frailty, a cluster of clinical and demographic factors including age and sex is associated with poor prognosis and mortality in elderly patients [7–12]. Although studies demonstrated an increased prognostic value of MPI according to sex and older age [13, 14], at present these factors are not included in this tool. Among clinical factors, systemic inflammation correlates with important clinical outcomes in older patients acutely hospitalized, including mortality, duration of hospital stay and nonelective readmissions [15]. Although increased plasma levels of cytokines, such as interleukin-6, have been correlated with short-term mortality in hospitalized older patient independently of frailty [16], their widespread use and applicability is limited by the availability of laboratory measurement. Therefore, alternative biomarkers easily obtainable in clinical practice should be identified and tested for mortality prediction. The Glasgow Prognostic Score (GPS), a simple tool derived from the combination of C-reactive protein and albumin levels [17], has been developed and validated to predict mortality in cancer patients and in conditions characterized by inflammation and/or malnutrition including COPD, relapse of idiopathic pulmonary fibrosis and myocardial infarction in the elderly [18–21].

Another useful assessment is that of hydration status, since dehydration is common in hospitalized older adults ranging from over one third to 50% of geriatric adults admitted to the hospital as emergencies [22, 23]. Impaired hydration is easily diagnosed by serum osmolality or alternatively by calculated serum osmolarity by validated formulas [24, 25]. Dehydration may develop quickly in those at increased risk with life-threatening consequences both in hospital after discharge [22, 26–28]. Rapid identification of this condition allows to establish a prompt treatment, which results in complete reversibility in mild and impending forms. Despite its high recurrence rates and poor prognosis, dehydration is often overlooked and it is not included within clinical algorithms developed for mortality prediction of older hospitalized patients.

Thus, the objectives of this study were: (1) to test the independent associations with short-, intermediate- and long-term mortality of inflammation, impaired hydration, and frailty adjusted for age and sex at hospital admission (2) to assess whether the addition of one or more variables independently affecting mortality can enhance the predictive performance of the MPI and its risk categories on 1-year mortality.

## Materials and methods

### Study design, setting and population

This was a retrospective observational study conducted in the Department of Medicine, Geriatric Unit, University Hospital of Trieste, Italy. This study was based on a secondary analysis of a prospectively collected database. All consecutive patients aged ≥ 65 years and admitted from January 1st 2019 to December 31st 2019 from the Emergency Department were considered for eligibility. For patients presenting more than one admission into the Geriatric Unit, only the first admission during the study period was considered. Patients with incomplete CGA assessment were excluded.

A minimum required sample size of 442–448 patients was calculated to detect, with a probability of a type I error of 0.05 and a type II error of 0.2, a 1-year mortality rate difference between MPI severe- and moderate-risk groups of approximately 30%, according to previous literature [14].

### Ethics

This study is part of the PROPOSING IDEAS (PROgnostic factors of Poor hospital Outcome based on clinical Severity, nutrItional and NursinG indexes IDEntified on AdmiSsion in elderly/frail patients) study, approved by the Regional Bioethics Committee of Friuli Venezia Giulia, Italy (Protocol number: 28217; date of approval: 31 July 2018). All the procedures performed in the study were in accordance with the Declaration of Helsinki and can be classified as “usual practice”. At hospital admission, all enrolled patients authorised the use of their clinical data for study purposes.

### Study variables

Clinical, functional and laboratory data assessed on admission were collected from the hospital electronic archive and from patients’ medical records.

The MPI was computed upon hospital admission based on the routinely collected Comprehensive Geriatric Assessment (CGA), comprising data on number of drugs at admission, cohabitation status, functional (by Basal and Instrumental Activities of Daily Living), cognitive (by Short Portable Mental Status Questionnaire) and nutritional status (by Mini Nutritional Assessment), risk of developing pressure sores (by Exton-Smith Scale) and comorbidities (by the Cumulative Illness Rating Scale). The MPI was thus calculated by including the information from the above-reported domains of the CGA according to the methodology previously reported, establishing three grades of MPI (range: 0 to 1): low risk (0.0–0.33); moderate risk (0.34–0.66); and severe risk (0.67–1.0) [29].

Serum osmolarity was calculated based on the equation by Krahn and Khajuria [30], validated in very different populations of older adults [31] (osmolarity = 1.86 × (Na^+^ + K^+^) + 1.15 × glucose + urea + 14; each component measured in mmol/L). Patients were considered dehydrated if calculated osmolarity was > 300 mmol/L, while a condition of impending dehydration was defined with an osmolarity of 296–300 mmol/L.

GPS was calculated based on the combination of C-reactive protein and albumin levels and the following categories were identified: 0 (good prognosis—CRP ≤ 10 mg/L and albumin ≥ 3.5 g/dL), 1 (intermediate prognosis—CRP > 10 mg/L and albumin ≥ 3.5 g/dL, or CRP ≤ 10 mg/L and albumin < 3.5 g/dL) and 2 (poor prognosis—CRP > 10 mg/L and albumin < 3.5 g/dL).

### Study end-points

The primary study endpoint was mortality—defined as death from any cause—within 12 months from hospital admission. Furthermore, hospital length of stay and all-cause mortality at 60 and 180 days were analyzed as secondary endpoints. Date of death was obtained from the public Regional electronic mortality register.

### Data analysis

Data distribution was evaluated using the Kolmogorov–Smirnov test. The difference between the means was analyzed using the unpaired Student *t*-test, after determining whether equal variance could be attributed to the subgroups according to Levene’s test. One-way analysis of variance (ANOVA) was applied for all comparisons between the subgroups. The nominal variables were described as a number and percentage, and analyzed with contingency tables and the *χ* test.

Five logistic regression models were tested to verify the extent of which the addition of covariates supposed to affect mortality independently from MPI (i.e.: age, sex, GPS, and serum osmolarity) affects the predictive power (that is, the ability to anticipate an outcome) of the MPI to predict 1-year patient mortality. Four categories (i.e.: ≤ 80; 81–85; 86–90; and > 90 years) were created according to 25th, 50th and 75th percentiles of the study population age. The following covariates were subsequently added to a simple logistic regression model (Model 1) consisting of MPI alone: age and sex (Model 2); age, sex and osmolarity (Model 3); age, sex and GPS (Model 4); and age, sex, GPS and osmolarity (Model 5). Results were reported as unadjusted an adjusted odds ratios (OR) with respective 95% confidence intervals (CI). The coefficient of the determination of each model was calculated based on the Nagelkerke *R*^2^. The fitted values (predicted probability) from the multivariable regression models were used to construct receiver operating characteristic (ROC) curves and to compare this with the ROC curve derived from unadjusted MPI. The performance of the above predictive models in discriminating between patients who would die or not after 1 year from hospital admission was tested by calculating the area under ROC curve (AUC), which results were interpreted according to the following criteria: 0.50–0.59: poor; 0.60–0.69: moderate; 0.70–0.79: good; 0.80–0.89: very good; and ≥ 0.90: excellent discrimination [32]. Since the performance of the predictive model could have been overestimated as simply established on the sample used to build it, the possible ‘over-optimism’ in the performance of the final model was tested by the bootstrapping technique, as this analysis was recommended for estimating internal validity of predictive logistic regression models [33]. Three-thousand replications of random sampling with replacement by drawing same size samples (i.e., 529 subjects) from the original data set were performed. The difference between the AUC obtained by the bootstrapping re-sampling method and the original AUC represented the optimism for the study data set.

Forward stepwise multivariate logistic regression models were run to test the predictive power of the MPI on patient mortality at different time intervals, controlled for the above covariates. Multivariate Cox regression models with forward stepwise selection were used as a sensitivity analysis to estimate the time-to-event effect of the MPI categories on the risk of death at the same time intervals. Results were reported as unadjusted an adjusted hazard ratios (HR) with respective 95% CI and adjusted survival curves.

All statistical analyses were performed using the software IBM SPSS Statistics, version 24.0 (Armonk, NY, US: IBM Corp.), while for the bootstrapping procedure a dedicated software was used [34]. For all tests, an alpha level of *p* ≤ 0.05 was set for statistical significance.

## Results

During the study period, 587 patients were admitted to the study ward. All patients had a complete MPI assessment on admission. After excluding subjects having more the one admission in the study period, 529 patients constituted the study population. For each, the complete outcome data were available. Table 1 shows the main characteristics of the study population. After excluding the patients who dead before hospital discharge, the hospital LOS for patients discharged alive from hospital (*n* = 526, 99.4%) was 10.9 ± 7.7 days.

**Table 1:** Main characteristics of the study population

Living condition	
Living alone	199 (37.6%)
Living with relatives	250 (47.3%)
Institutionalized	80 (15.1%)
Main medical diagnosis^a^	
Anemia	380 (71.8%)
Infection/sepsis	347 (65.6%)
Heart failure	298 (56.3%)
Fall	295 (55.9%)
Atrial fibrillation	262 (49.5%)
Diabetes	225 (42.5%)
Cancer	210 (39.7%)
Obesity	196 (37.1%)
Dementia	185 (35.0%)
Kidney failure	149 (28.2%)
Constipation	137 (25.9%)
Chronic obstructive pulmonary disease	115 (21.7%)
Urinary tract infection	63 (11.9%)
Stroke	58 (11.0%)
Liver failure	40 (7.6%)
Body Mass Index (kg/m^2^)	23.8 ± 5.3
Comprehensive Geriatric Assessment	
Cumulative Illness Rating Scale	4.6 ± 2.0
Exton Smith Scale	15.6 ± 3.7
Basal Activities of Daily Living	3.0 ± 2.3
Instrumental Activities of Daily Living	3.6 ± 3.1
Short Portable Mental Status Questionnaire	3.3 ± 3.3
Mini Nutritional Assessment-Short Form	19.3 ± 6.4
Regularly used drugs (*n*)	5.7 ± 3.1
Hospital outcome	
Discharged to home	318 (60.1%)
Discharged to a healthcare facility	201 (38.0%)
Transferred to other hospital department	7 (1.3%)
Dead	3 (0.6%)

^a^Each individual patients may have more than one diagnosis

In the bivariate analysis, MPI was higher (*p* < 0.001) for patients who died within 1 year from hospital admission (*n* = 196; 0.60 ± 0.20) compared to those who survived (*n* = 333; 0.44 ± 0.20). Moreover, 1-year mortality was significantly related to higher GPS, osmolarity and age and male sex (Table 2). No statistically significant difference in hospital LOS was found for patients presenting a severe-risk MPI compared to low- or moderate-risk (*p* = 0.658), while the LOS was significantly longer for patients with higher GPS (GPS < 2: 9.1 ± 6.9 days; GPS = 2: 12.8 ± 8.4 days; *p* < 0.001) and osmolarity (osmolarity ≤ 300: 10.3 ± 6.5 days; osmolarity > 300: 12.2 ± 10.0 days; *p* = 0.043).

**Table 2:** Main characteristics of the study population and distribution according to 1-year mortality

Variable	All patients *n* = 529	Survived *n* = 333	Dead *n* = 196	*p *value
Age (years)	84.6 ± 7.3	83.2 ± 7.0	86.9 ± 7.1	< 0.001
≤ 80	147; 27.8	111; 75.5%	36; 24.5%	< 0.001
81–85	130; 24.6	97; 74.6%	33; 25.4%	
86–90	132; 25.0	73; 55.3%	59; 44.7%	
> 90	120; 22.7	52; 43.3%	68; 56.7%	
Sex				
Female	326; 61.6%	217; 66.6%	109; 33.4%	0.029
Male	203; 38.4%	116; 57.1%	87; 42.9%	
Multidimensional Prognostic Index	0.50 ± 0.22	0.44 ± 0.20	0.60 ± 0.20	< 0.001
Low risk (0–0.33)	150; 28.4	119; 79.3%	31; 20.7%	< 0.001
Moderate risk (0.34–0.66)	225; 42.5	155; 68.9%	70; 31.1%	
Severe risk (0.67–1)	154; 29.1	59; 38.3%	95; 61.7%	
Glasgow Prognostic Score*				
0	85; 17.1%	69; 81.2%	16; 18.8%	< 0.001
1	144; 17.2%	103; 71.5%	41; 28.5%	
2	252; 47.6%	135; 53.6%	117; 46.4%	
Serum osmolarity (mmol/L)	295.1 ± 13.6	293.2 ± 13.4	298.4 ± 13.3	< 0.001
≤ 295	274; 51.8	193; 70.4%	81; 29.6%	0.001
296–300	104; 19.7	60; 57.7%	44; 42.3%	
> 300	151; 28.5	80; 53.0%	71; 47.0%	

Data are presented as: “mean ± standard deviation” or “number; percentage”. *n = 481

The logistic regression models (Table 2) showed that only severe-risk MPI category maintains its power as a predictive tool on 1-year mortality in the fully adjusted final model. Moreover, the performance of severe-risk MPI category falls (OR from 6.2 to 4.1) when it was adjusted for the considered variables. The unadjusted MPI showed a moderate discrimination power (AUC 0.678; 95% CI 0.628–0.729; *p* < 0.001) at separating those patients who died from those who survived after 1 year since hospital admission. When adjusted for sex, age, GPS and/or serum osmolarity, the discrimination power became good in all explored models, with a progressive increase of AUC with the transition from Model 2 to Model 5. More in detail, when compared to Model 1 the addition of the above predictors increased the explained variance by 47.9%, 59.0%, 66.7% and 80.6% in Models 2, 3, 4 and 5, respectively, while the discrimination power increased by 8.3%, 9.3%, 10.6% and 12.5% in the more complete predictive models, respectively (Table 3).

**Table 3:** Logistic regression models to predict 1-year mortality

Model	AUC (95% CI); *p* value	*R*^2^; *p* value	Variables included in the final model	Adjusted^*^ OR (95% CI); *p* value
1	0.678 (0.628–0.729); < 0.001	0.144; < 0.001	MPI—moderate risk (0.34–0.66)^†^	1.734 (1.067–2.818); 0.026
			MPI—severe risk (0.67–1.00)^†^	6.181 (3.706–10.310); < 0.001
2	0.734 (0.688–0.781); < 0.001	0.213; < 0.001	MPI—severe risk (0.67–1.00)^†^	5.213 (2.997–9.069); < 0.001
			Age 86–90 years^‡^	2.185 (1.266–3.772); 0.005
			Age > 90 years^‡^	2.814 (1.578–5.016); < 0.001
			Sex (male)^¥^	2.081 (1.384–3.128); < 0.001
3	0.741 (0.695–0.788); < 0.001	0.229; < 0.001	MPI—severe risk (0.67–1.00)^†^	4.980 (2.847–8.709); < 0.001
			Age 86–90 years^‡^	2.099 (1.210–3.643); 0.008
			Age > 90 years^‡^	2.775 (1.550–4.967); 0.001
			Sex (male)^¥^	1.977 (1.310–2.984); 0.001
			Serum osmolarity > 300 mOsm/L^§^	1.741 (1.108–2.736); 0.016
4	0.750 (0.706–0.795); < 0.001	0.240; < 0.001	MPI—severe risk (0.67–1.00)^†^	4.468 (2.479–8.054); < 0.001
			Age 86–90 years^‡^	2.000 (1.127–3.551); 0.018
			Age > 90 years^‡^	2.700 (1.462–4.986); 0.002
			Sex (male)^¥^	1.822 (1.179–2.816); 0.007
			GPS = 2^¶^	2.214 (1.165–4.205); 0.015
5	0.763 (0.719–0.807); < 0.001	0.260; < 0.001	MPI—severe risk (0.67–1.00)^†^	4.133 (2.273–7.516); < 0.001
			Age 86–90 years^‡^	1.846 (1.031–3.306); 0.039
			Age > 90 years^‡^	2.589 (1.394–4.807); 0.003
			Sex (male)^¥^	1.705 (1.097–2.649); 0.018
			Serum osmolarity > 300 mOsm/L^§^	2.069 (1.272–3.364); 0.003
			GPS = 2^¶^	2.483 (1.289–4.780); 0.007

*AUC* area under receiver operating characteristic curve, *CI* confidence interval, *MPI* Multidimensional Prognostic Index, *GPS* Glasgow Prognostic Score

^*^Except for model 1 (unadjusted OR)

^†^Reference: MPI low risk (0.00–0.33)

^‡^Reference: age ≤ 80 years

^¥^Reference: female sex

^§^Reference: serum osmolarity ≤ 295 mOsm/L

^¶^Reference: GPS = 0 points

**Fig. 1:**
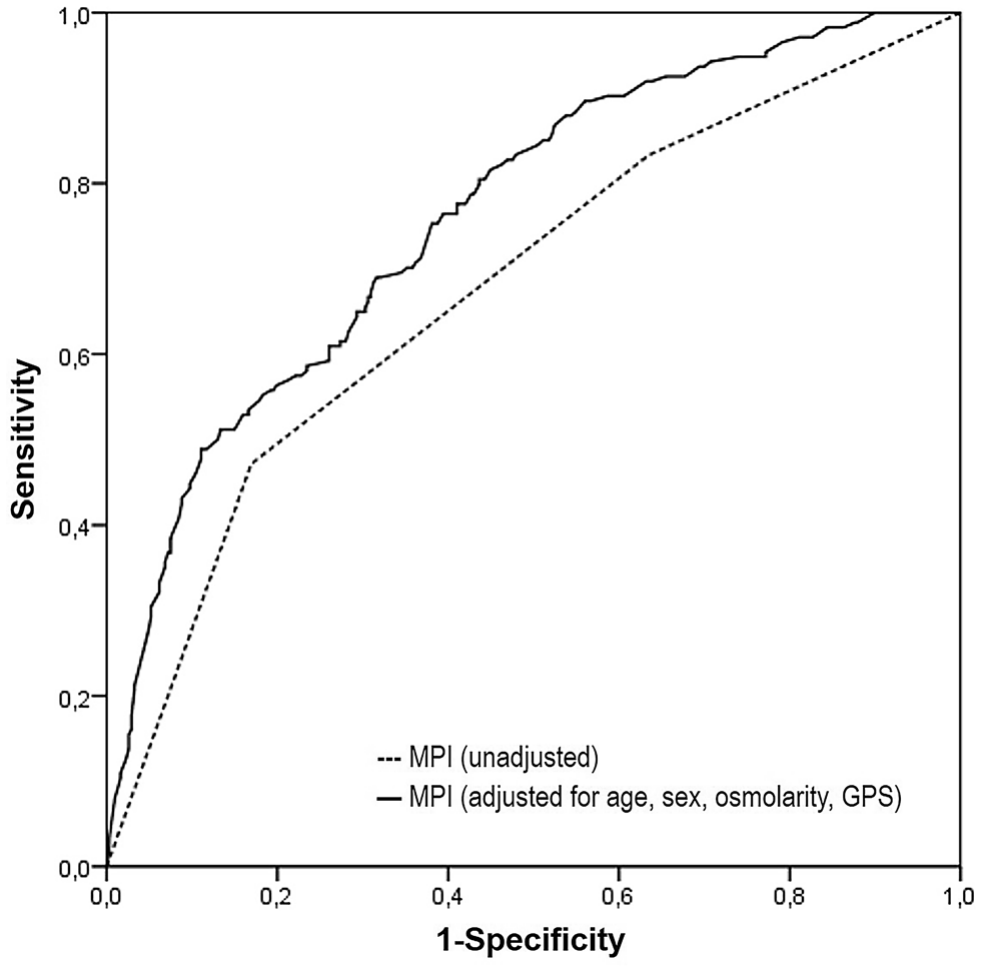
Comparison of area under the curve (ROC) curves for unadjusted and adjusted multidimensional prognostic index (MPI)

The best discrimination power in identifying patients who were at risk for 1-year mortality was demonstrated by Model 5 (AUC 0.763; 95% CI 0.719–0.807; *p* < 0.001). Figure 1 shows the ROCs for the unadjusted and fully adjusted logistic models. The bootstrap optimism estimate was 0.002; thus, the AUC corrected for optimism was 0.761 (95% CI 0.724–0.798), showing a good internal validity of the regression model.

**Fig. 2:**
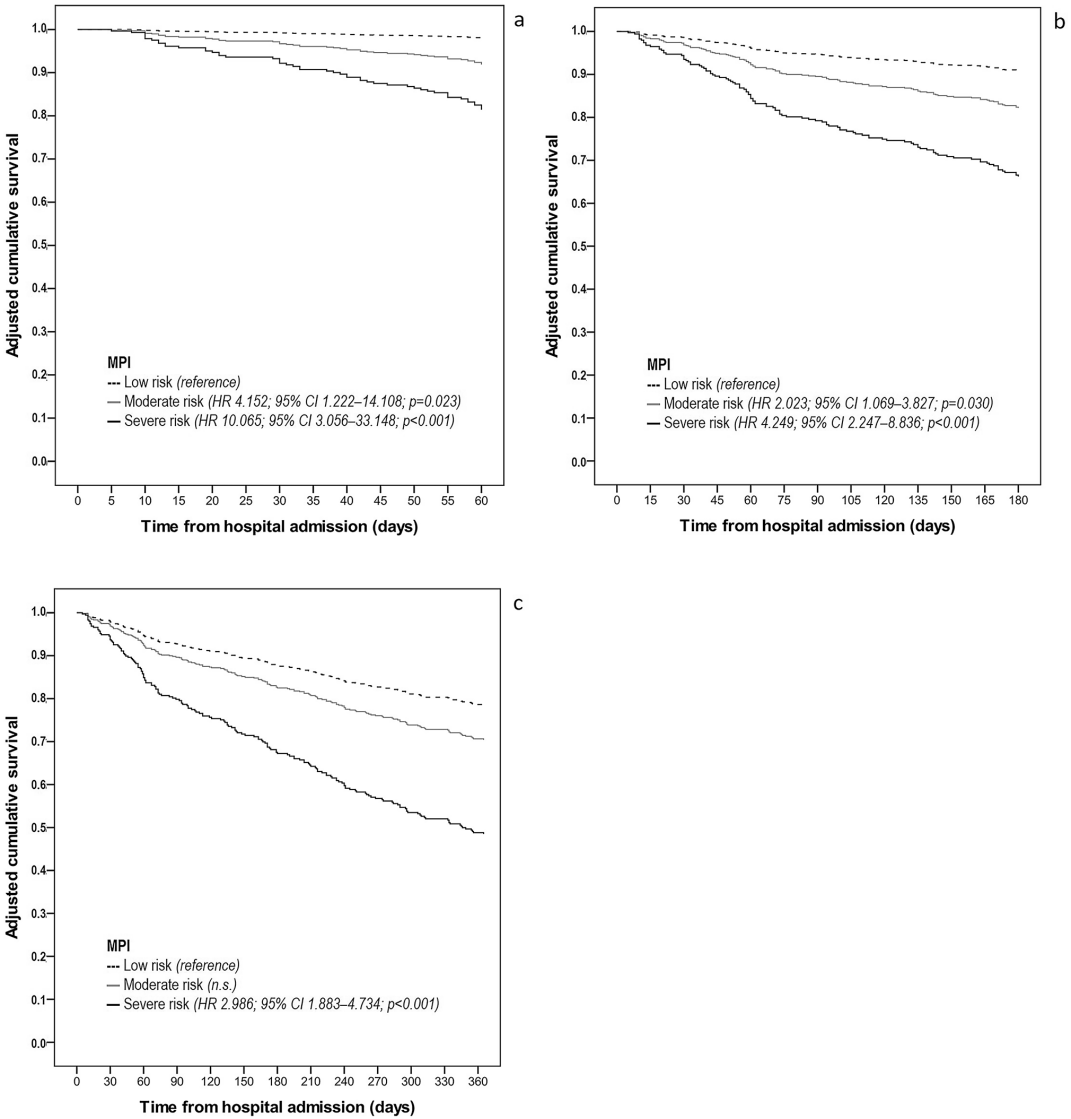
Adjusted survival curve (multivariate Cox regression analysis) at 60 days (**a**), 180 days (**b**) and 365 days (**c**) from hospital admission for patients belonging to different multidimensional prognostic index (MPI) categories

The cumulative mortality rates were 11.2% (*n* = 59), 23.4% (*n* = 124) and 37.1% (*n* = 196) after 60, 180 and 365 days since hospital admission, respectively. The multivariate logistic regression analysis (Table 4) showed that severe risk MPI class (> 0.66) on admission was an independent predictor of mortality in all considered time intervals, while a similar predictive power was demonstrated for the moderate risk MPI class (0.33–0.66) only at 2 and 6 months. GPS-2 showed to be significantly associated with mortality at 180 and 365 days, with a trend toward an increased risk of death at 60-days (OR 3.393; *p* = 0.051). Among the other considered covariates, male gender and age > 90 years were predictive for both 180 and 365 days mortality, while at the latter time-point also age 85–90 years and current dehydration as measured by increased serum osmolarity resulted independently associated with mortality. The sensitivity analysis carried out via multivariate Cox regression models showed that MPI had a similar trend as an independent predictor of mortality in all considered time intervals, although the HRs were lower than the ORs found by fully adjusted logistic regression models (Fig. 2).

**Table 4:** Stepwise multiple logistic regression analysis of mortality at different time interval from hospital admission on study variables

	60-day mortality OR (95% CI); *p* value	180-day mortality OR (95% CI); *p* value	365-day mortality OR (95% CI); *p* value
MPI—low risk	*Reference*	*Reference*	*Reference*
MPI—moderate risk	4.309 (1.237–15.009); 0.022	2.133 (1.062–4.286); 0.033	*n.s*
MPI—severe risk	11.464 (3.373–38.958); < 0.001	5.408 (2.632–11.111); < 0.001	4.133 (2.273–7.516); < 0.001
GPS = 0	*Reference*	*Reference*	*Reference*
GPS = 1	*n.s*	*n.s*	*n.s*
GPS = 2	3.393 (0.992–11.602); 0.051	3.010 (1.275–7.107); 0.012	2.483 (1.289–4.780); 0.007
Sex (male)	*n.s*	1.809 (1.109–2.950); 0.017	1.705 (1.097–2.649); 0.018
Age (≤ 80 years)	*n.s*	*Reference*	*Reference*
81–85 years	*n.s*	*n.s*	*n.s*
86–90 years	*n.s*	*n.s*	1.846 (1.031–3.306); 0.039
> 90 years	*n.s*	2.470 (1.250–4.881); 0.009	2.589 (1.394–4.807); 0.003
Euhydration	*n.s*	*n.s*	*Reference*
Impending dehydration	*n.s*	*n.s*	*n.s*
Current dehydration	*n.s*	*n.s*	2.069 (1.272–3.364); 0.003

*OR* odds ratio, *CI* confidence interval, *MPI* Multidimensional Prognostic Index, *GPS* Glasgow Prognostic Score, *n.s.* nonsignificant

## Discussion

In this study, we showed that—in addition to MPI moderate/high risk—severe inflammation as assessed by GPS-2 is independently associated with short/intermediate and long-term mortality in elderly patients acutely hospitalized, while demographic factors (age and gender) and hydration status are significant predictors only at longer time-points. In addition, the comprehensive model including severity of inflammation and frailty, age and sex significantly improves predictive accuracy of MPI alone.

The importance of inflammation and frailty in the management of older adults affected by acute diseases has been advocated by substantial literature [35, 36]. Accordingly, in this retrospective study we found a significant association between higher inflammation (as defined by GPS-2), severe frailty (MPI risk score > 0.66) and greater intermediate- (6 months) and long-term mortality (1 year) in older patients acutely hospitalized.

Systemic inflammation is a feature of many acute conditions and is a strong predictor of adverse prognosis in adult and older patients [15, 37]. The aging process is associated to low-grade, systemic inflammation and dysregulation of many inflammatory pathways (the so called “inflammaging”) [38]. These conditions are related to the occurrence of sarcopenia, insulin resistance, cardiovascular events and neurodegenerative disorders [39]. Moreover, systemic inflammation has been reported to play an important role in the prognosis of older patients [40, 41], and increased levels of selected biomarkers, especially IL-6, have been shown to correlate with higher short-term mortality after hospitalization [16]. Here we extend these findings by showing that severity of inflammation measured at hospital admission is a good prognostic factor in the intermediate and long term, while in the short-term statistical significance was borderline (*p* = 0.051) possibly because of a significant number of missing data. In addition, our findings demonstrate that the Glasgow Prognostic Score is a valuable and clinically relevant parameter with prognostic implications. Previous studies have shown a strong association between albumin levels measured at admission and poor outcomes in older adults [42]. However, albumin is not considered a specific marker of inflammation as it also reflects nutritional status. The widespread use and applicability of more specific markers of inflammation is hampered by their availability in the clinical setting [16], therefore alternative biomarkers easily obtainable should be tested for predictivity. The Glasgow Prognostic Score, a simple tool based on routinely collected laboratory parameters, is easily computable and—by considering both serum albumin and CRP—might better reflect the impact of systemic inflammation on outcomes than one single variable. Available evidence on the short-term mortality predictive power of CRP in geriatric patients acutely hospitalized is controversial [15, 16]. Our results are consistent with the hypothesis that frailty is associated with higher inflammation, in line with previous studies reporting a correlation between increased serum levels of proinflammatory cytokines and frailty [36]. We believe that providing a model that incorporates GPS measured at hospital admission to the frailty score is of particular interest in terms of intermediate- and long-term risk stratification and management of frail older patients with acute conditions.

This study confirms the predictive reliability of MPI at all tested time-points, with the highest predictivity in the high-risk class. Since age and sex are biologically important factors in the association between mortality and frailty and the prognostic value of MPI becomes progressively higher when these variables are included in the model [14], we adjusted the predictivity analysis including these covariates. Our findings confirm an independent role for age and male sex on mortality as the impact of age increased progressively being maximal at 1 year for patients > 85 years old. These results are consistent with previous studies showing that age and inflammation are associated in the development of age-related diseases and frailty [36, 43] and that mortality of older adults at any given frailty severity score is sex-dependent, with male gender showing higher mortality risk than the female counterpart [11]. The association between male sex and mortality became significant at 6 months and 1 year, but not earlier, in line with a previous study which failed to demonstrate a male–female health survival paradox in hospitalized older adults for 28-day mortality [44]. The combined model including MPI and GPS corrected by age and sex had incremental prognostic value for mortality at 1 year compared with MPI alone. Compared to MPI alone (AUC 0.678), the AUC from multivariate regression models for predicting mortality increased when age and sex (AUC 0.734) and then GPS (AUC 0.750) were added (Table 2).

Another finding of interest is that impaired hydration was not associated with short- and intermediate-term mortality, but only with long-term mortality with an osmolarity cut-off > 300 mOsm/L indicating current dehydration. The lack of association between earlier mortality and dehydration can be explained by the clinical characteristics of the cohort, since only patients with mild acute diseases were admitted to the Geriatric Unit, while those with critical conditions including severe dehydration due to decompensated diabetes, acute kidney failure and water-loss hypernatremia were admitted to other hospital wards. Nonetheless, current dehydration was associated with long-term mortality and its addition to the comprehensive prognostic model increased its accuracy (AUC 0.763). Although a potential correlation between dehydration and specific domains of frailty including polypharmacy, functional decline, cognitive impairment and malnutrition has been suggested [45, 46], this link has not been fully demonstrated by available literature [23, 47]. The results of this study suggest that dehydration diagnosed at hospital admission is a predictive marker (although less robust than GPS, frailty and age) of long-term mortality and might therefore be taken into account when considering 1-year mortality especially in high-risk patients.

Our findings might have important clinical consequences. All of the variables included in the model are easily obtainable and have proven to increase the long-term predictive accuracy of MPI. Inflammation and impaired hydration diagnosed at admission are potentially modifiable risk factors for severe outcomes and their management might provide important benefits for long-term survival. Unfortunately, interventional studies on the long-term effects of targeting inflammation and incident dehydration are missing. Future studies addressing these important issues are required.

The present investigation has strengths and limitations. A point of strength is that this study is one of the few assessing the role of inflammation, current dehydration and frailty on short- and long-term mortality in older adults. Since the confirmed internal validity of the final logistic regression model, out findings may be considered reliable in similar populations. However, our results should be considered in the light of some limitations. First the research was performed in a single Unit of a University Hospital, and the results may not be extended to patients treated in other hospitals or clinical units. Second, the database included patients with mild acute diseases, therefore the findings might not apply to patients with conditions at higher risk of lethality. Third, a shorter mortality endpoint (e.g., 30 days) was not considered in the analyses due to an insufficient number of death events in this time-span, precluding to appropriately build the multivariable regression models. Therefore, our results should be confirmed by replicating the study across different populations and with larger samples.

In conclusion, inflammation as scored by GPS-2 and MPI-severe risk were predictive for a higher risk of mortality in the short to long term in acutely hospitalized older patients. Current dehydration, male sex and age > 85 years were associated with increased risk of mortality only in the mid and/or long term. Since demographic, inflammation and hydration biomarkers are not included in the MPI—which in this way may not represent completely enough the risk of death—the inclusion of these variables might increase the prognostic accuracy of the MPI in clinical practice when assessed on hospital admission. Diagnosing and targeting inflammation and dehydration both in hospital and after discharge might carry beneficial effects for early- and long-term survival. Further studies are needed to confirm this hypothesis.

## Data Availability

On request.
